# Ameliorative Effects of Escin on Inflammation via Glucocorticoid Receptor (GR) in Atopic Dermatitis (AD) Mouse Model

**DOI:** 10.4014/jmb.2410.10025

**Published:** 2025-03-11

**Authors:** A Yeon Park, Jung Ok Lee, You Na Jang, Su-Young Kim, Ji Hye Heo, Yu-Jin Kim, Jung Min Lee, Dae Won Yoon, Beom Joon Kim, Joon Seok

**Affiliations:** 1 Department of Medicine, Graduate School, Chung-Ang University, Seoul 06973, Republic of Korea; 2 Department of Dermatology, College of Medicine, Chung-Ang University Hospital, Seoul 06974, Republic of Korea

**Keywords:** Escin, atopic dermatitis (AD), filaggrin, thymic stromal lymphopoietin (TSLP), inflammation, skin barrier, glucocorticoid receptor (GR)

## Abstract

Atopic dermatitis (AD) is a chronic, relapsing inflammatory skin disease characterized by intense itching. Escin, derived from *Aesculus hippocastanum*, has several pharmacological functions, including anti-inflammatory and anti-viral effects, and exhibits glucocorticoid-like actions in inflammatory responses. However, its impact on AD has not been extensively studied. We investigated the anti-inflammatory effects of escin on AD and elucidate its underlying mechanism of actions in the dermatophagoides farinae extract (DFE)-induced AD mouse model. The AD-induced group treated with escin showed a significant reduction in immunoglobulin E (IgE) levels, ear thickness, epidermal thickness, and mast cell infiltration compared to the AD group. Additionally, escin significantly reduced the dermatitis score and the sizes of the spleen and lymph nodes. Notably, escin inhibited the reduction of filaggrin expression induced by DFE, while suppressing the upregulation of thymic stromal lymphopoietin (TSLP), interleukin (IL)-4, IL-13, IL-1β, and tumor necrosis factor (TNF)-α. Escin also significantly suppressed DFE-induced NF-κB expression. Interestingly, pre-treatment with RU486, a glucocorticoid receptor (GR) antagonist, attenuated the therapeutic effects of escin. In line with these findings, escin modulated the IFN-γ/TNF-α-mediated changes in TSLP and filaggrin expression in HaCaT keratinocyte cells. Furthermore, escin inhibited the lipopolysaccharide (LPS)-induced overproduction of nitric oxide (NO), protein expression of inducible nitric oxide synthase (iNOS) and cyclooxygenase-2 (COX-2), and mRNA expression of IL-6 and IL-1β in RAW 264.7 cells. These results indicate that escin may offer therapeutic potential in treating AD through the GR.

## Introduction

Atopic dermatitis (AD) is chronic, relapsing, and intensely itchy inflammatory skin disease. In both Europe and the United States, up to 20% of children and 7-14% of adults are affected by AD [1]. The prevalence of AD is unfortunately increasing [2]. The primary symptoms of AD, such as xerosis and severe pruritus, substantially affect the quality of life for individuals suffering from the condition [3]. The development of AD is attributed to structural and immunological defects in the epidermal barrier, imbalance of the skin microbiome, genetic predisposition, and environmental factors [4]. An increased expression of Th2 cytokines, including interleukin (IL)-4, IL-5, and IL-13, contributes to the disruption of the skin barrier in AD-affected skin [5]. Elevated serum immunoglobulin (Ig) E levels promote histamine release from skin mast cells, thereby aggravating AD [6].

The skin, consisting of three primary layers - the epidermis, dermis, and subcutaneous layer - serves a crucial protective function, with the epidermis serving as the body's initial barrier [7, 8]. Filaggrin (FLG) is crucial for both the structure and function of the epidermis, influencing skin barrier and acts as the body's initial defense against pathogens, pollutants, and allergens. Therefore, FLG deficiency significantly increased the skin permeability to allergens and pathogens [9, 10]. Keratinocytes, the predominant cells in the epidermis, play a crucial role in promoting AD proinflammatory condition and secrete thymic stromal lymphopoietin (TSLP).

TSLP activates dendritic cells (DCs) and mast cells, thereby triggering Th2-type immune responses, and is regarded as a key molecule in the pathophysiology of AD [11]. TSLP levels in AD patients are significantly higher compared to those in healthy individuals. Specially, TSLP downregulates FLG expression, thereby contributing to the pathogenesis of AD [12].

Currently, standard treatments for AD include topical anti-inflammatory drugs such as corticosteroids (*e.g.*, glucocorticoids (GCs)), calcineurin inhibitors (*e.g.*, tacrolimus), and anti-histamines [13, 14]. Among them, GCs are commonly used pharmacologically to treat inflammatory conditions due to their potent anti-inflammatory properties [15, 16]. The glucocorticoid receptor (GR) is a nuclear receptor that mediates the physiological effects of GC hormones. GR functions as a ligand-dependent transcription factor, inhibiting the production of NF-kB and various pro-inflammatory cytokines, including IL-1, IL-2, IL-6, IL-8, cyclooxygenase-2 (COX-2), prostaglandins (PGs), interferon‐gamma (INF-g), and tumor necrosis factor alpha (TNF-α) [17-19]. Although GCs provide temporary relief by reducing inflammation, prolonged use can lead to side effects such as localized skin thinning, stinging, itching, and a burning sensation[20]. As result, there is an increasing demand for novel biological therapies that are both effective and safe for the treatment of AD.

Escin, which is derived from *Aesculus hippocastanum*, is a natural mixture of pentacyclic triterpenoid saponins, celebrated for its therapeutic versatility [21]. Escin exhibits various effects including anti-edematous, anticancer, anti-inflammatory, and anti-allergic activities [22-25]. Interestingly, Luca Gallelli *et al*. demonstrated that escin exerts anti-inflammatory efficacy through the GR, while minimizing the side effects associated with glucocorticoids [26].

To date, while the anti-inflammatory properties of escin have been extensively studied such as edema, and arthritis, its potential impact on AD, particularly through the GR pathway, remains largely unexplored. Therefore, in this study, we investigated the anti-AD effects on escin using RU486, a GR antagonist, in a *Dermatophagoides farina* extracts (DFE)-induced AD mouse model. Additionally, we examined its anti-inflammatory effects and its potential to enhance skin barrier function in RAW 264.7 murine macrophage cells and HaCaT human keratinocyte cells.

## Materials and Methods

### Chemicals and Reagents

Sigma (USA) supplied the following products: lipopolysaccharide (LPS, L6529), dexamethasone (Dexa) (purity>97%, D4902), RU486 (purity ≥ 98%, M8046), escin (purity ≥ 95%, E1378), and carboxymethylcellulose, sodium salt (CMC). TNF-α and IFN-γ recombinant proteins were acquired by R&D Systems (USA). Fetal bovine serum (FBS) and Dulbeccós Modified Eagle Medium (DMEM), among other cell culture chemicals, were acquired from WelGENE (Republic of Korea).

### Cell Culture and Treatment

The Korean Cell Line Bank (KCLB) in Seoul, Korea is where the RAW 264.7 mouse macrophage cell was acquired. Furthermore, the HaCaT human keratinocyte was acquired from Cell Lines Services GmbH (Germany). The cells were grown in DMEM with 100 U/ml penicillin, and 100 μg/ml streptomycin and 10% Fetal Bovine Serum (FBS). The cells were kept in an incubator maintained at 37°C and with 5% CO_2_ in a humid atmosphere. During the incubation period, the medium was changed every two to three days. Cells were pretreated for 2 h with 0.3, 1, or 3 μg/ml of escin or 0.5 μg/ml of Dexa. Subsequently, 20 ng/ml of TNF-α/IFN-γ or 1 μg/ml of LPS was administered, and the cells were incubated for 30 min or 24 h. Dimethyl sulfoxide (DMSO, Sigma) was used to first dilute escin to a 50% concentration, and DMEM was then used to serially dilute it to 0.01%.

### Cell Viability Assay

HaCaT and RAW264.7 cells were cultured in 96-well plates at a density of 5×10^3^ cells/well and 1×10^5^ cells/well respectively and were exposed to different doses of escin (0, 0.3, 1, 3, 10 or 0, 1, 2, 4, 6, 8, 10 μg/ml). After 24 h, the cells were incubated in a medium containing WST-8 solution (QuantiMax TM, Biomax, Republic of Korea) for 2 h at 37°C with 5% CO_2_. The absorbance was measured at 450 nm using a SpectraMax 340 spectrophotometer (Molecular Devices, USA).

### Measurement of Nitric Oxide (NO) Concentration

After being pretreated for 2 h with escin at doses of 1 or 3 μg/ml, the RAW264.7 cells were treated for 24 h with 1 μg/ml LPS. The cell culture medium was centrifuged and the supernatants were collected. NO levels were analyzed using a Nitrate Plus Detection kit (cat. no. 21023) from iNtRON Biotechnology (Republic of Korea) following the manufacturer’s instructions.

### Reverse Transcription Followed by Quantitative PCR (RT-qPCR)

The RAW264.7 cells were subjected to a 2 h pre-treatment with escin at 1 or 3 μg/ml, followed by a 30-min treatment with 1 μg/ml of LPS. Using the TRIzol reagent (Invitrogen, USA), total RNA was extracted. Reverse transcription was used to synthesis single-strand cDNA using PrimeScript TM RT Master Mix (Takara Bio, Japan). Utilizing qPCR PreMIX SYBR-Green (Enzynomics, Republic of Korea) and a CFX96 thermocycler (Bio-Rad Laboratories, USA), the resultant cDNA was subjected to qPCR. The thermal cycle settings included a 15 min initial activation step at 95°C, which was followed by a 45-cycle cycle of denaturation for 10 sec at 95°C, annealing for 15 sec at 60°C, and elongation for 30 sec at 72°C. Using the 2^-ΔΔCt^ quantification approach, gene expression levels were computed as a cycle threshold (Ct) value and normalized to that of glyceraldehyde-3-phosphate dehydrogenase (GAPDH) [27]. Table 1 contains a list of the primers used in the qPCR.

### Immunocytochemistry (ICC)

The cells were subjected to a 30 min fixation process using 4% paraformaldehyde (PFA), followed by washing with a phosphate-buffered saline (PBS). They were then blocked using 3% bovine serum albumin (BSA) and 0.2%Triton X 100 in PBS at room temperature (RT) for 1 h. Finally, they were incubated with primary antibodies against GR (1:3000, Cell Signaling Technology, USA) for an entire night at 4°C. Following a PBS wash, the cells were incubated for 1 h at RT in the dark with anti-rabbit IgG FITC secondary antibodies (MA, 1: 3,000, Abcam, UK). A confocal microscope (LSM 880, Zeiss AG) was used to view the stained cells after the cell nuclei were counterstained with 4’, 6-diamidino-2-phenylindole (DAPI, ImmunoBioScience Corp.) at RT for 30 min.

### Western Blot Analysis

RIPA lysis buffer (Thermo Fisher Scientific, USA) was used to extract the skin and cell protein, and the Bradford reagent (Sigma) was used to measure the protein concentration. On an 8% sodium dodecyl sulfate poly acrylamide (SDS-PAGE) gel, equal volumes of protein (10 μg) were separated and then transferred to nitrocellulose membranes (Cytiva, UK). After that, the membranes were probed overnight at 4°C using primary antibodies against GR (Cell Signaling Technology, 1:5000, 3660s), NF-kB (Cell Signaling Technology, 1:5000, 8242s), TSLP (Abcam, 1:5000, ab188766), Filaggrin (Thermo Fisher Scientific, 1:5000, PA5-116911), COX-2 (Cell Signaling Technology, 1:5000,12282s), and iNOS (Cell Signaling Technology, 1:5000, PA1-038). The blocking process was carried out in 5% skim milk in Tris-buffered saline containing 0.1% Tween-20 (TBS-T). The membrane was then treated for 1h at RT with HRP-conjugated anti-mouse (PI-2000-1, 1:5000, Vector Labs, USA) or anti-rabbit (PI-1000-1, 1:5000, Vector Labs) secondary antibodies. Immunodetection was performed using an Amersham ECL kit (GE Healthcare, USA), following the manufacturer’s protocol. A ChemiDoc MP Imaging System (Bio-Rad Laboratories) was used to observe the protein bands, and Image J V 1.8.0 (NIH Image, USA) was utilized for analysis, normalizing all target proteins to β-actin.

### Animal Experimental Design

**Fig. 1:**
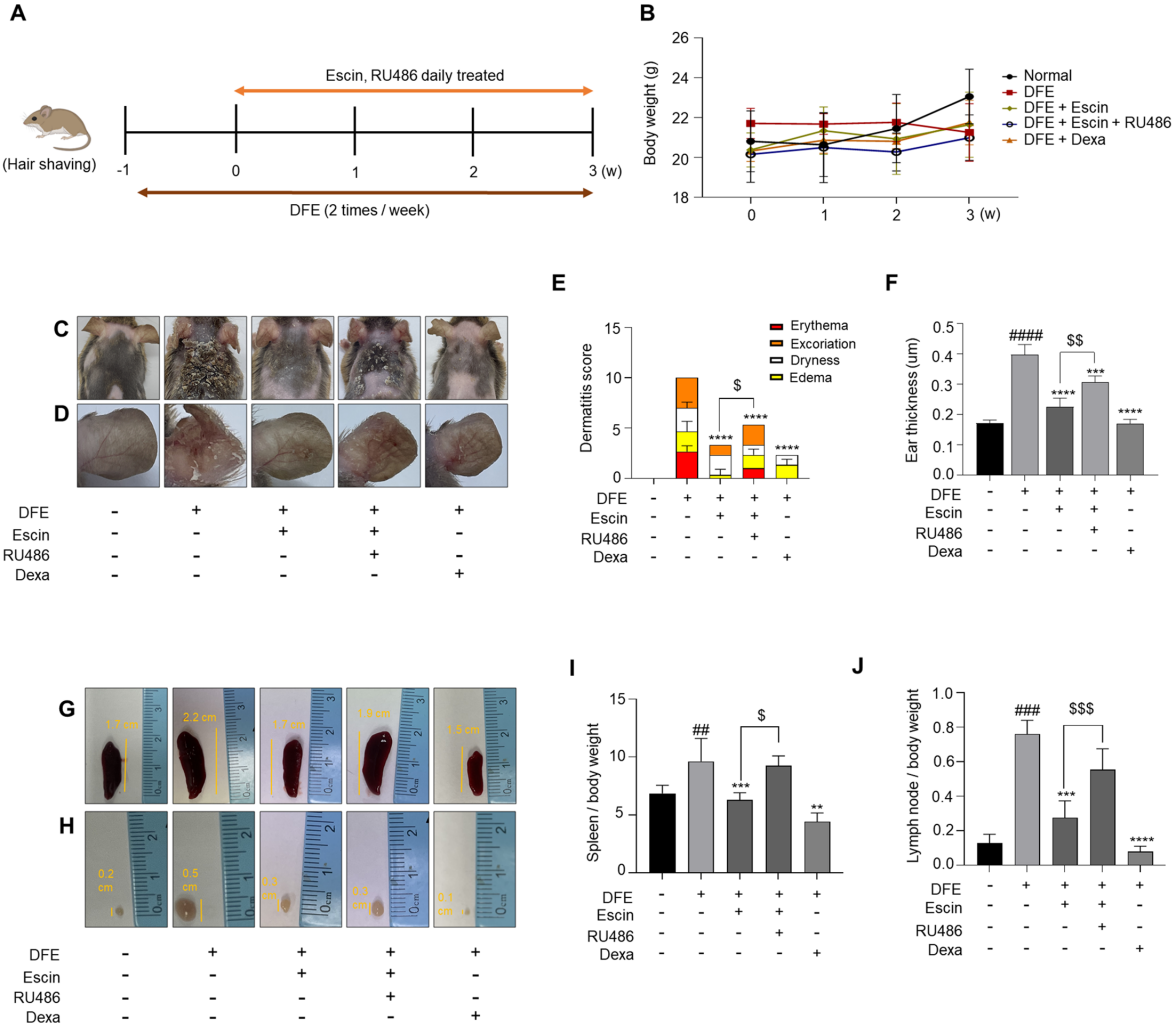
Escin alleviates DFE-induced AD skin symptoms through the GR. (**A**) Experimental design. (**B**) Body weight. (**C**) Representative photographs of mouse dorsal skin and (**D**) Ear. (**E**) Dermatitis score was analyzed as the sum of scores for each symptom. (**F**) Ear thickness. (**G-J**) Representative images used to compare the size and weight of the spleen and lymph node. Organ/whole body weight. The results are expressed as the mean ± SEM (*n* = 5– 6 per group). ^##^, *p* < 0.01; ^###^, *p* < 0.001; ^####^, *p* < 0.0001 compared with the normal group. **, *p* < 0.01; ***, *p* < 0.001; ****, *p* < 0.0001 compared with the DFE-treated group. ^$^, *p* < 0.05; ^$$^, *p* < 0.01; ^$$$^, *p* < 0.001 compared with the DFE + escin +RU486group.

From Saeron Bio (Saeronbio Inc., Republic of Korea), A total of 25 female NC/Nga mice (age, 6wks; weight, 17–20 g; *n* = 5 per group) were acquired (Charles River Laboratories, Japan). The mice experiments were conducted in accordance with the principles of laboratory animal care of the National Institutes of Health (NIH, USA). The Institutional Review Board and Chung-Ang University's animal experiment ethics committee approved every experiment before it was carried out (IRB Approval No. 202301020095). The mice were housed in a 12-h light/12-h dark cycle with the following parameters for one week: 23 ± 2°C, 55 ± 10% humidity. The mice were divided into 5 groups at random, with 5 mice in each group: group 1, normal; group 2, DFE only; group 3, DFE + escin (10 mg/kg); group 4, DFE + escin (10 mg/kg) + RU486 (10 mg/kg), a GR antagonist (Sigma); group 5, DFE + Dexa (5 mg/kg). After resting for 1week, 4% SDS was sprayed onto the shaved skin, and DFE (Biostir, Japan) was topically applied to the skin twice a wk for 3 wks. Dexamethasone (Dexa) and escin were dissolved in CMC at concentrations of 5 mg/kg and 10 mg/kg, respectively, and were administered orally on daily basis. RU486, at a concentration of 10 mg/kg, was dissolved in CMC and administered daily through oral gavage (Fig. 1A).

### Measurement of Dermatitis Severity

The anesthetic was diluted (10-fold) with normal saline and comprised of Zoletil (0.008 cc/10 g; 40 mg/kg) and Rompun (0.002 cc/10 g; 5 mg/kg). The dorsal skin was photographed in proximity using a digital single-lens reflex camera (D5200; Nikon, Japan). The severity of AD-like dorsal skin lesions was assessed by dermatitis score at the end of the experimental period modified versions of previously published criteria [27]. The dorsal skin severity scores were recorded for AD mice based on four skin symptoms (excoriation/erosion, scaling/dryness, edema, erythema/hemorrhage). The scoring range indicators were 0 (none), 1 (mild), 2 (moderate) and 3 (severe). Clinical skin score was defined as the sum of the individual scores with a maximum score of 12.

### Sample Preparation

Prior to blood collection, the mice, with an average body weight of 22 ± 4 g, were anesthetized using 2% isoflurane in oxygen in an anesthetic chamber for induction and 1.5% isoflurane for maintaining anesthesia. Blood samples of 800 μl each were collected via retro-orbital bleeding into heparinized tubes. The sera were subsequently separated by centrifugation at 1,500 ×*g* for 10 min and stored at −80°C until further analysis. The mice were euthanized using CO_2_ asphyxiation prior to performing biopsies of the skin, spleen, and lymph nodes. In detail, the mice were transferred to a new cage and immediately euthanized by displacing the air with 100% CO_2_ at a flow rate of 30% of the chamber volume per minute, completing the procedure within 5 min [28, 29]. In this study, humane endpoints were established to minimize suffering; animals were closely monitored for signs of distress, and any showing significant pain or discomfort were promptly euthanized.

### Body Weight, Spleen Weight, and Lymph Node Size

Throughout the study, the micés weights were recorded once a week. The size of the axillary lymph nodes and the weight of the spleen were assessed at the conclusion of the study. Each mousés overall weight was used to standardize the weight of the spleen.

### Measurement of Serum IgE and Cytokines in Mouse Skin

Mini Collect tubes (Greiner bio-one, Austria) were used to separate serum samples from whole blood. Utilizing an ELISA kit (88-50-460-88, Invitrogen), the amount of IgE in the serum was determined. To extract proteins, 30 mg of regional skin was homogenized in 600 μl of RIPA buffer. For 20 min, homogenates were centrifuged at 20,000 ×*g*. Using the Bradford reagent (Sigma), the total protein concentration in the supernatants was determined and equalized in each sample. An ELISA kit (Invitrogen) was used to measure the levels of IL-1β (88-7013-22), TNF-α (88-7324-88), TSLP (88-7490-22), IL-4 (88-7044-22), and IL-13(88-7137-22) in accordance with the manufacturer's instructions.

### Histological Analysis

Skin biopsies were preserved for 24 h in 10% formalin. Sections that were 5 μm thick and fixed in paraffin were cut, then placed on POLYSINE Slides from Thermo Fisher Scientific, dewaxed in xylene, and finally dried with an ethanol series. The thickness of the skin was examined using hematoxylin and eosin (H&E) staining. The purpose of toluidine blue (TB) staining was to see the cutaneous mast cells. Furthermore, primary antibodies against TSLP (Abcam, 1:200) and Filaggrin (GeneTex, 1:300) were used to stain tissue slides. 0.05 % PBS-Tween 20 was used to wash the slides before they were treated with Vector Laboratories' 3,3-diaminobenzidine (DAB) chromogenic substrate. The slides were cleaned, dried, and then mounted with Thermo Fisher Scientific's Permount mounting media. A slide scanner (Pannoramic MIDI; 3DHISTECH Ltd., Hungary) was used to take pictures of all the stained tissue slides, and Case Viewer software (V 2.7; 3DHISTECH Ltd., Hungary) was used for analysis.

### Statistical Analysis

The data is shown as the average ± standard deviation (SD) of three or more separate studies. Unpaired one-way analysis of variance (ANOVA) was used to analyze the data, and it was followed by the Bonferroni post hoc test. Software called GraphPad Prism 9.0 (GraphPad Software, USA) was used to do statistical analysis. At least three replications of each experiment were conducted. *p* < 0.05 was considered to indicate a statistically significant difference.

## Results

### Escin Alleviates AD Skin Symptoms through the GR in DFE-Treated NC/Nga Mice

We investigated the role of Escin in DFE-induced AD-like mice using Dexa as a positive control. The experimental schedule is presented in Fig. 1A. Throughout the experiment, there were no statistically significant differences in the mice weights among all groups (*p* < 0.05) (Fig. 1B). Photographs of the dorsal skin and ear of mice confirmed that escin significantly alleviated atopic clinical symptoms in these areas. However, the positive effects of escin on AD symptoms were abolished by RU486, a GR antagonist (Fig. 1C and 1D). To comprehensively analyze the effects of escin on AD symptoms, clinical features of AD, including erythema excoriation, dryness, edema, and excoriation, were evaluated and scored. The severity score was markedly decreased in the escin-treated group, while RU486 pre-treatment led to a significantly higher severity score compared to escin treatment (Fig. 1E). Additionally, the ear thickness of escin-treated mice was lower than that of DFE-treated mice, whereas RU486 pre-treatment blocked these effects of escin. (Fig. 1F). To comprehensively assess atopic symptoms in the mouse model, the spleen and local lymph nodes were examined. DFE-mediated inflammation led to spleen and lymph nodes enlargement, which was mitigated by escin treatment (Fig. 1G-1H). However, pre-treatment with RU486 suppressed the effect of escin on the increase in spleen and lymph node weights induced by DFE (Fig. 1I and 1J). These findings suggest that escin alleviates clinical symptoms of AD via the GR.

### Escin Decreases Epidermal Thickness and Mast Cell Infiltration, while Adjusting the Expression of TSLP and Filaggrin to Mitigate Skin Barrier Disruption

**Fig. 2:**
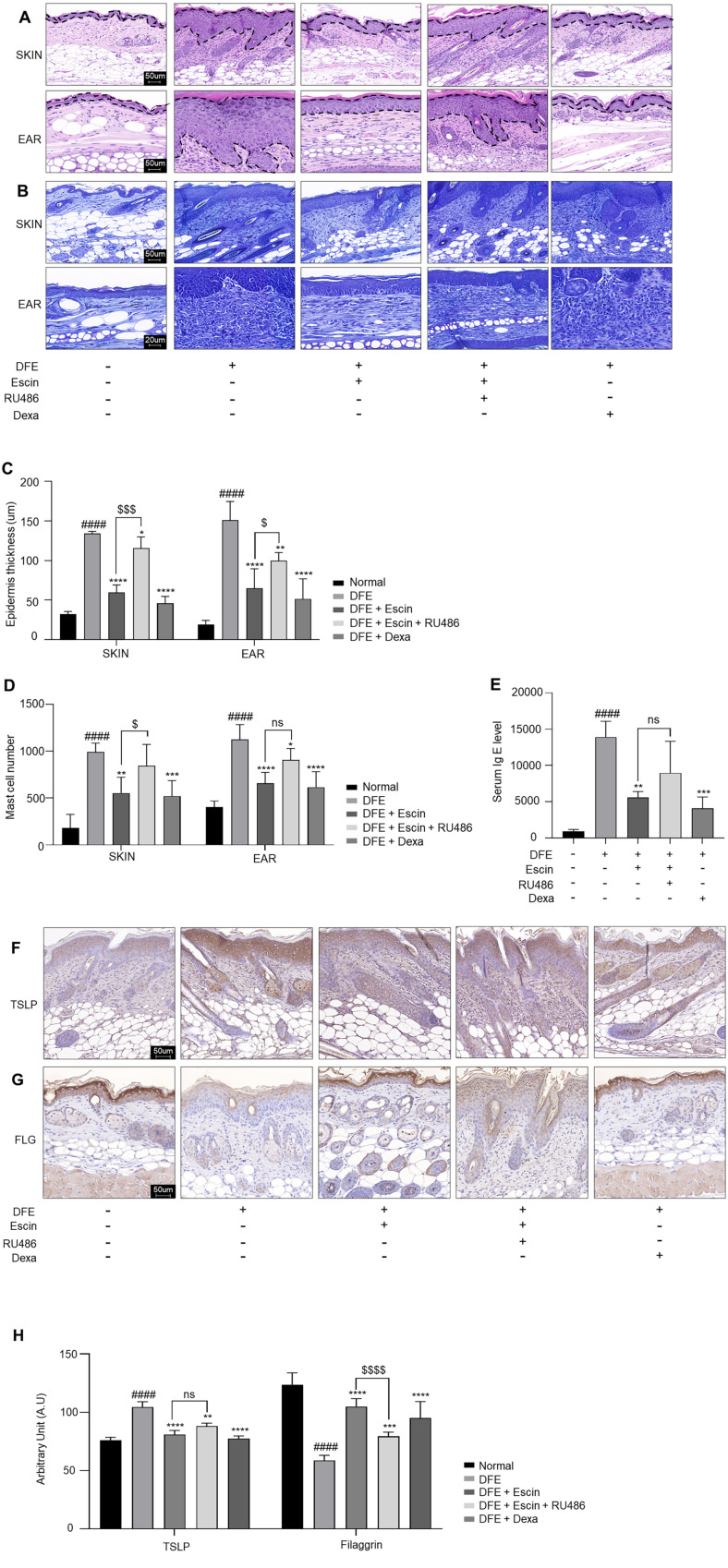
Escin affects histological features in the dorsal tissue of DFE-induced AD NC/Nga mice and positively regulates the expression of TSLP and Filaggrin via GR. (**A**) Representative images of H&E and (**B**) TB staining of the dorsal skin and ear tissue of the mice (scale bars, 50 μm, 20 μm). (**C**) Epidermal thickness in H&E stained sections. (**D**) Mast cells were counted in toluidine stained sections. (**E**) Serum Ig E levels measured by ELISA in DFE-treated NC/Nga mice. (**F**) TSLP (**G**) Filaggrin immunohistochemistry. 50μm scale bar. (**H**) Immunohistochemistry arbitrary units (a.u.) of TSLP, Filaggrin. The results are expressed as the mean ± SEM (*n* = 5–6 per group). ^###^, *p* < 0.001; ^####^, *p* < 0.0001 compared with the normal group. *, *p* < 0.05; **, *p* < 0.01; ***, *p* < 0.001; ****, *p* < 0.0001 compared with the DFE-treated group. ^$^, *p* < 0.05; ^$$$$^, *p* < 0.0001 compared with the DFE + escin + RU486 group.

The epidermal thickness was significantly greater in the DFE-treated group compared to the control group. However, escin reduced the DFE-induced epidermal thickness (Fig. 2A and 2C). The number of TB-stained mast cells in the dermis significantly decreased in the escin-treated group compared to the DFE-treated group. However, RU486 pre-treatment reversed the escin-induced decrease in mast cell infiltration (Fig. 2B and 2D). Serum IgE levels in DFE-treated mice were elevated 15-fold relative to those in control mice (Fig. 2E). This increase was inhibited by escin, whereas pre-treatment with RU486 reversed this effect. These results suggest that escin inhibits the increase in epidermal thickness, mast cell infiltration, and IgE production induced by DFE through the GR.

We assessed the effect of escin on TSLP and filaggrin expression using IHC staining. TSLP expression was elevated in the DFE-treated group, but escin inhibited the DFE-induced increase in TSLP, and pre-treatment with RU486 attenuated the effect of escin (Fig. 2F and 2H). Furthermore, compared to controls, DFE reduced filaggrin expression in the epidermis, whereas escin treatment reversed this reduction and restored filaggrin levels. Consistently, RU486 treatment blocked these effects (Fig. 2G and 2H). We found that escin protects skin barrier from damage by regulating the expression of TSLP and filaggrin by GR.

### Escin Exerts Anti-Inflammatory Effects by Increasing GR and Decreasing NF-κB Expression

**Fig. 3:**
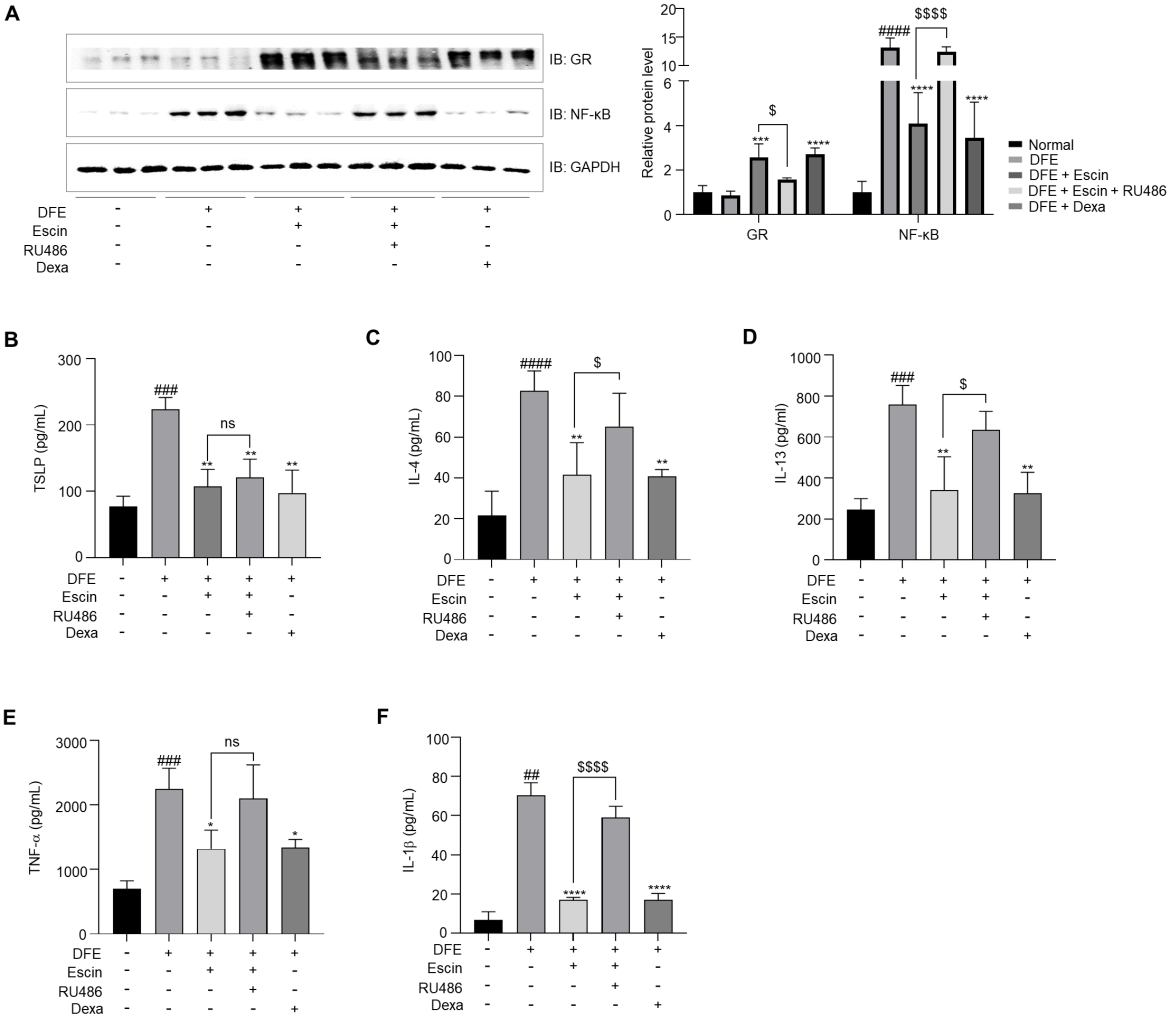
Escin exerts anti-inflammatory effects through the GR in DFE-treated AD NC/Nga mice. (**A**) Protein levels of GR and NF-kB in the skin of DFE-treated NC/Nga mice were examined using western blot analysis. Cytokine (**B**) TSLP, (**C**) IL-4, (**D**) IL-13, (**E**) TNF-α, (**F**) IL-1β levels measured by ELISA in DFE-treated NC/Nga mice. The results are expressed as the mean ± SEM (*n* = 4–5 per group). ^##^, *p* < 0.01; ^###^, *p* < 0.001; ^####^, *p* < 0.0001 compared with the normal group. *, *p* < 0.05; **, *p* < 0.01; ****, *p* < 0.0001 compared with the DFE-treated group. ^$^, *p* < 0.05; ^$$^, *p* < 0.01, ^$$$$^, *p* < 0.0001 compared with the DFE + escin + RU486 group.

To assess whether escin activates GR in the AD mice model, we examined GR expression in the dorsal skin. The levels of GR significantly increased in the escin-treated group compared to the control and DFE groups. However, pre-treatment with RU486 reversed this trend. Furthermore, the elevated NF-κB levels in DFE group were reduced by escin, whereas pre-treatment with RU486 resulted in NF-κB expression comparable to those observed in the DFE-treated group (Fig. 3A). We also measured inflammatory cytokines using ELISA. TSLP levels decreased from 223.49 ± 17.95 pg/ml to 107.62 ± 25.54 pg/ml in the DFE-treated group and escin-treated group. However, this reduction was reversed by RU486 pre-treatment (Fig. 3B). Escin also decreased the levels of IL-4, IL-13, TNF-α, and IL-1β, but these effects were notably reversed by RU486 pre-treatment (Fig. 3C-3F). Based on these results, we confirmed that escin suppresses the expression of upregulated inflammatory cytokines associated with AD via GR.

### Escin Affects TSLP and Filaggrin Expression in HaCaT Cells and Exerts Anti-Inflammatory Functions in LPS-Treated RAW 264.7 Cells

**Fig. 4:**
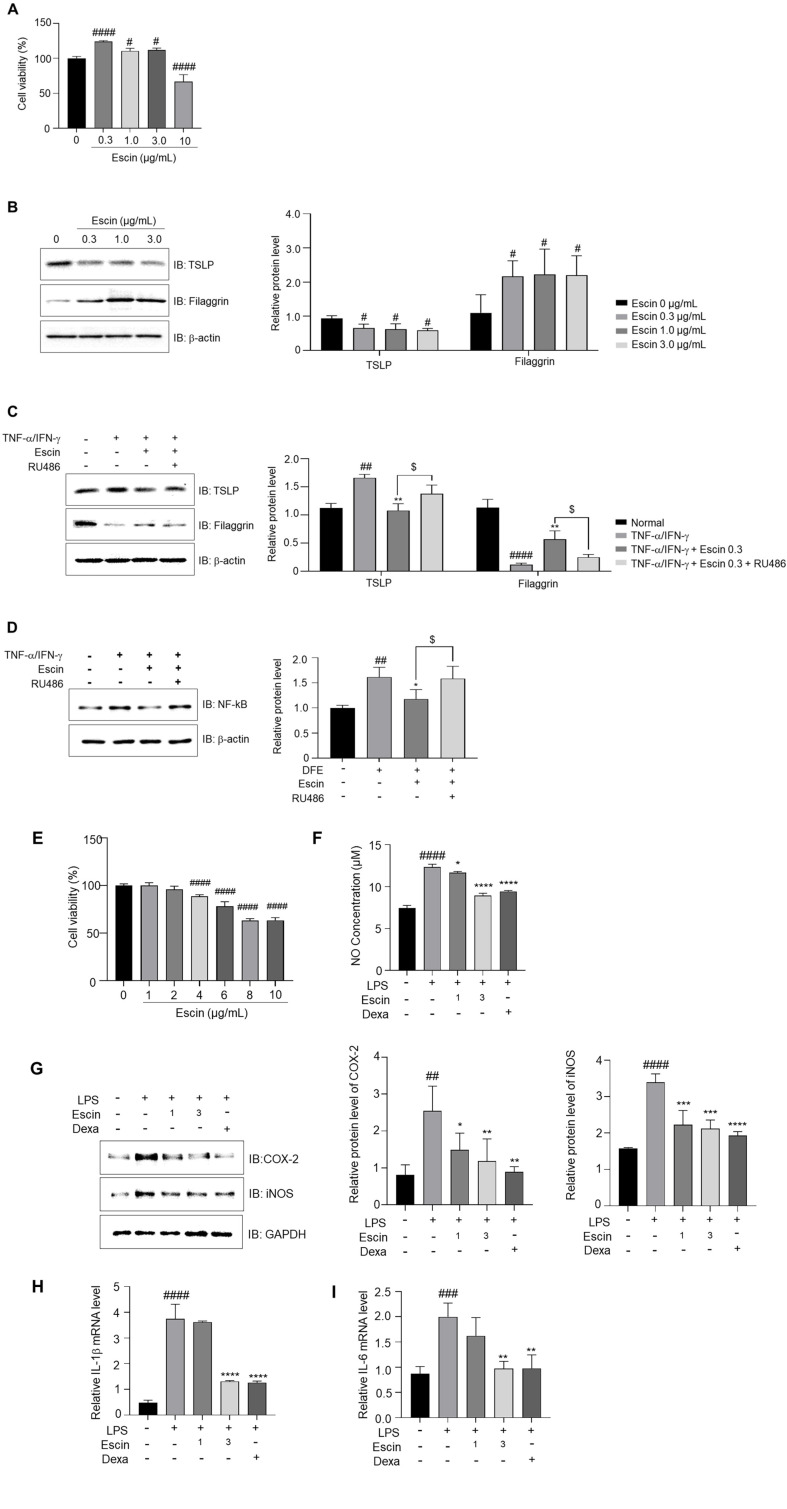
Escin positively regulates the expression of TSLP and Filaggrin via GR in TNF-α/IFN-γ treated HaCaT cells and functions in the anti-inflammatory response in LPS-treated RAW 264.7 cells. (**A**) Using the WST 8 cell viability assay, HaCaT cells were treated with escin (0, 0.3, 1.0, 3.0, and 10 μg/ml) for a duration of 24 h. (**B**) TSLP and Filaggrin in HaCaT cell treated with Escin only (0, 0.3, 1.0, 3.0 μg/ml) for 24 h. (**C**) The expression of TSLP and Filaggrin after co-treatment with TNF-α/IFN-γ and escin, RU486 for 24 h. (**D**) The expression of NF-kB after treatment with TNF-α/IFN-γ and escin, RU486 for 24 h. (**E**) WST-8 assay was used to determine the viability of RAW 264.7 cells. (**F**) NO concentration in LPS-treated RAW 264.7 cells. (**G**) Protein levels of COX-2 and iNOS in LPS or escin-treated RAW 264.7 cells. (**H**) IL-1β and (**I**) IL-6 in LPS or escin-treated RAW 264.7 cells. The cells were pre-treated with escin (E; 1, 2, 4, 6, 8, 10 μg/ml or F-I; 1, 3 μg/ml) for 2 h and then exposed to LPS. Following incubation for 24 h (**A-G**) or 30 min (**H–I**), the cells were examined by RT-qPCR, western blot, or ELISA. The protein levels in the western blot analysis were measured and shown in relation to the β-actin levels. The mean ± standard deviation represents the results. ^#^, *p* < 0.05; ^##^, *p* < 0.01; ^###^, *p* < 0.001; ^####^, *p* < 0.0001 compared with the normal group. *, *p* < 0.05; **, *p* < 0.01; ***, *p* < 0.001; ****, *p* < 0.0001 compared with the TNF-α/IFN-γ treated group or LPS-treated group. $, *p* < 0.05 compared with the TNF-α/IFN-γ + escin + RU486 group.

Escin promoted the proliferation of HaCaT cells at concentration of 0.3 to 3 μg/ml. Escin (10 μg/ml) was cytotoxic (Fig. 4A). Escin reduced TSLP expression while simultaneously increasing filaggrin expression (Fig. 4B). Treatment with TNF-α/IFN-γ in HaCaT cells induced an increase in TSLP expression and a decrease in filaggrin expression, which were reversed by escin treatment. However, pre-treatment with RU486 prevented this effect (Fig. 4C). In consistently, NF-κB expression was also conversed by RU486 (Fig. 4D). Taken all together, it demonstrates that escin regulate the expression of TSLP and filaggrin via GR in HaCaT cells. Escin demonstrated cytotoxicity at concentrations of 4 μg/ml and higher in RAW 264.7 cells (Fig. 4E). Based on these results, escin (1 and 3 μg/ml) were selected for further analysis. Escin effectively suppressed the LPS-induced NO secretion (Fig. 4F). In addition, escin inhibited the increase in iNOS and COX-2 expression mediated by LPS (Fig. 4G). It also significantly suppressed the expression of LPS-induced IL-1β and IL-6 (Fig. 4H and 4I). These results indicate that escin has anti-inflammatory activity in LPS-treated RAW 264.7 macrophage cells.

## Discussion

In this study, we investigated the anti-inflammatory activity of escin on AD via GR signaling pathway. To achieve this goal, we employed RU486, a potent GR antagonist in DFE induced AD mice. House dust mites are a significant risk factor for allergic diseases, including AD and asthma [30]. Allergens from *Dermatophagoides farinae* commonly trigger allergic diseases and play a role in the pathogenesis of human AD. Consequently, a DFE-induced AD animal model is more effective in replicating human AD [31, 32]. Firstly, we found that escin markedly improved AD symptoms in DFE-induced AD mice, including, reducing dorsal skin and ear lesions, as well as alleviating clinical characteristics such as excoriation, dryness, edema, and erythema. However, these effects were reversed by pre-treatment with RU486 (Fig. 1C-1J), suggesting that the anti-inflammatory effects of escin on AD are associated with the GR.

Pruritus negatively impacts the quality of life for patients with AD. Therefore, the primary therapeutic goal is to alleviate pruritus symptoms and improve the quality of life [33]. Mast cells secrete histamine which triggers allergy symptoms, such as itching and swelling when exposed to external antigens or bacteria [34]. As shown in Fig. 2B and 2D, escin significantly reduced the number and infiltration of mast cell as shown by TB staining. These results demonstrate that escin is effective in alleviating AD symptoms by alleviating pruritus. However, this study only demonstrated a reduction in plasma IgE levels (Fig. 2E). To further validate the itch-relieving effects of escin, it is essential to confirm its impact on histamine levels.

The GR is a GC-dependent transcription factor and regulates the expression of numerous genes involved in metabolism, immune function, stress response, and other physiological processes [35]. GR is composed of three functional domains: (1) an amino (N)-terminal transcriptional activation domain (NTD), (2) a central DNA binding domain (DBD), and (3) a C-terminal ligand-binding domain (LBD) [36]. Upon binding with glucocorticoids, the GR translocates to the nucleus, where it forms homodimers and associates with glucocorticoid response elements (GREs) in the promoter regions of target genes, thereby modulating their expression either positively or negatively [37]. It is well known ligand-bound GR can inhibit the activity of immunogenic transcription factors, including nuclear NF-κB, AP-1, and T-bet, leading to a marked reduction in inflammation [38]. As shown in Fig. 3A, escin increased the expression of GR while simultaneously decreasing the expression of NF-κB. However, these effects were attenuated by prior-treatment with RU486. These results indicate that escin exerts its effects on AD by inhibiting NF-κB expression via GR pathway. Notably, inflammation of AD closely related to Th1/Th2 lymphocytes. NF-κB is involved in the production of IL-4, IL-5, and IL-13, which play a role in AD [39]. As shown in Fig. 3C, the reduced expression of IL-4 and IL-13 induced by escin treatment suggests that it modulates the dominant Th2 immune response, restoring balance and alleviating atopic inflammation.

Zhao shu-qi *et al*. suggested that escin has the possible involvement of GR to the anti-inflammation activity [40]. Consistently, we found that escin increased the expression of GR while simultaneously decreasing the expression of NF-κB on AD like inflammatory condition.

Furthermore, to further confirm whether the anti-inflammatory effects of escin on AD are modulated via GR pathway, we performed experiments using a RU486, GR antagonist in *in vivo*. Additionally, the anti-inflammatory effects of escin were demonstrated in RAW 264.7 macrophages. These anti-inflammatory effects of escin were also suppressed by RU486 in macrophages, indicating that escin exerts its anti-inflammatory effects through GR. Taken all together, we found that escin functions via GR. However, additional research is needed to investigate how escin decrease NF-κB expression. Generally, GC down regulate the expression of proinflammatory genes by two kinds modes. Firstly, GCs mitigate inflammatory responses through the induction of IkB. The other is that GC prevents the inflammatory reaction is by inhibition of NF-κB transcription factor activity, which was occurred through direct protein-protein interactions [41]. In future studies, we will investigate the mechanism by which escin inhibits NF-κB.

Keratinocytes are the most abundant epithelial cells in the skin. As an *in vitro* model, HaCaT keratinocytes are commonly employed due to their ease of use and their efficiency in yielding potentially significant results [42]. Treatment with TNF-α/IFN-γ in HaCaT keratinocytes is frequently used to model AD-like responses and to evaluate the effectiveness of functional foods or pharmaceuticals [43, 44]. In Figure 4C. the stimulation of TNF-α and IFN-γ in HaCaT cells results in the expression of TSLP. However, escin inhibits induction of TSLP mediated with TNF-α and IFN-γ treatment. In consistent with in skin of DFE-induced AD in NC/Nga mice, RU486 blocked escin-mediated TSLP down regulation. These results strongly suggested that escin suppress AD-induced TSLP expression and it mediated to anti-inflammatory effect on AD.

Macrophages contribute to chronic inflammation, such as in AD, by sustaining prolonged pro-inflammatory activity and impairing antigen presentation [45]. As shown in Figure 4F and 4H-4I-4I, escin significantly reduced NO, IL-1b and IL-6 LPS-induced RAW 264.7 macrophage cells, suggesting that escin has anti-inflammatory activity to the LPS-induced inflammation. The anti-inflammatory effect of escin plays a crucial role in improving AD. Conclusively, these effects suggest escin holds promising potential as a therapeutically effective agent for treating AD.

## Figures and Tables

**Table 1:** Primer sequences used for quantification of gene expression.

Gene		Primer sequence (5'→ 3')
Mouse IL-1β	F	GCACTACAGGCTCCGAGATGAA
	R	GTCGTTGCTTGGTTCTCCTTGT
Mouse IL-6	F	CTTGGGACTGATGCTGGTGACA
	R	GCCTCCGACTTGTGAAGTGGTA
Mouse β-actin	F	AGAGGGAAATCGTGCGTGAC
	R	CAATAGTGACCTGGCCGT
